# Pain management in infant immunisation: A cross-sectional survey of UK primary care nurses

**DOI:** 10.1017/S146342362300066X

**Published:** 2023-12-21

**Authors:** Annie P. Mabbott, Helen Bedford

**Affiliations:** University College London Great Ormond Street Institute of Child Health, London, UK

**Keywords:** Immunisation, nursing, paediatrics, pain, primary care, vaccination

## Abstract

**Background:**

Childhood immunisation is a critically important public health initiative. However, since most vaccines are administered by injection, it is associated with considerable pain and distress. Despite evidence demonstrating the efficacy of various pain management strategies, the frequency with which these are used during routine infant vaccinations in UK practice is unknown.

**Aim:**

This study aimed to explore primary care practice nurses’ (PNs) use of evidence-based pain management strategies during infant immunisation, as well as barriers to evidence-based practice.

**Methods:**

A questionnaire was developed and distributed to nurses throughout the UK via convenience sampling in paper and online formats. Questions assessed the frequency of pain management intervention use during infant immunisation and barriers to their use.

**Findings:**

A total of 255 questionnaire responses were received. Over 90% (*n* = 226) of respondents never used topical anaesthetics or sweet solutions during immunisations, while 41.9% advised breastfeeding occasionally (*n =* 103). Parent-/caregiver-led distraction was the most frequently used intervention, with most nurses using it occasionally (47.9%, *n =* 116) or often (30.6%, *n =* 74). Most practices had no immunisation pain management policy (81.1%, *n =* 184), and most PNs’ previous training had not included pain management (86.9%, *n =* 186). Barriers to intervention use included lack of time, knowledge and resources. Excluding distraction, pain management strategies were infrequently or never used during infant immunisation. Key barriers to using evidence-based strategies were lack of time, knowledge and resources.

## Background

Childhood vaccination is a highly effective and safe intervention which prevents millions of deaths and complications from serious infections each year (World Health Organization (WHO), 2023). However, since most vaccines are administered by intramuscular injection, it is also the most common cause of iatrogenic pain and distress in infancy. This has implications potentially lasting across the lifespan (McMurtry *et al*., 2015). Not only is unmitigated injection pain distressing for the infant, parent/caregiver and health professional at the time of vaccination, it may result in the development of fear of needles leading to future non-compliance with scheduled vaccines, as well as non-compliance or anxiety associated with preventive and therapeutic health care more generally. Fear of needles is common in children and in adults (McLenon and Rogers, 2019), and a recent estimate based on a systematic review of its prevalence as a barrier to vaccination reported rates of 5–13% among the general paediatric population and 8–28% among under vaccinated populations (Taddio *et al.*, 2022). This risk factor for vaccine hesitancy potentially increases the frequency or severity of outbreaks of vaccine-preventable diseases (Taddio *et al.*, 2015). Immunisation pain should therefore be considered a universal public health issue.

### Infant immunisation in the UK

The number of vaccines included in the UK routine schedule has increased over time. Where possible, vaccines are given as a combination formulation to reduce the number of injections, and since 1992, further antigens (*Haemophilus Influenzae* type b, Inactivated Polio Vaccine and Hepatitis B) have been added to the 3-in-1 Diphtheria, Tetanus, Pertussis (DTP) combination with a 6-in-1 vaccine now currently used in the UK (DTaP/IPV/Hib/HepB) (UK Health Security Agency (UKHSA), 2022). However, some new vaccines are given as separate injections. For example, Meningococcal B (MenB) vaccine was introduced in 2015 and is given at eight weeks, 16 weeks and one year (UKHSA, 2022). While representing important advances in public health, increasing the number of vaccines and thus injections given at any one visit may be a concern for parents/caregivers and health professionals (Wallace *et al.*, 2014).

Parental concern that vaccines are painful is one of the most common barriers to childhood vaccination (Mills *et al.*, 2005). In the UK, there are limited data regarding parental views about childhood immunisation pain, although a 2007 survey study of 859 English parents found 57% of parents would not want their child to have more than two injections per visit; the main reason reported was that fewer injections would be less painful (Bedford and Lansley, 2007). More recently in a UKHSA study of almost 1500 parents of children under four years of age, 27% of 688 parents who expressed some dissatisfaction with their most recent vaccination experience cited concerns about pain, distress or side effects caused by the vaccine (UKHSA, 2023). A recent UK study reported injection fears among adults to be common and the possible explanation for approximately 10% of COVID-19 vaccine hesitancy (Freeman *et al.*, 2023). Although direct evidence is currently lacking about the contribution of fear of needles or pain as a barrier to childhood vaccination in the UK, this does not nullify the importance of seizing all opportunities to reduce immunisation pain. Yet given its importance, it attracts relatively little attention. None of the major sources of vaccine recommendations and training in the UK address the issue in detail; in 2020, a small amount information about pain management for infant vaccination was added to the Health Education England e-learning programme (Health Education England, 2023), and the NHS provides some limited information on preparing children for vaccination designed for parents/carers (NHS, 2023).

Vaccine uptake in the UK in infancy is generally high, although no vaccines met the 95% target in 2021–2022 (NHS Digital, 2022); 92.3% of 12-month-old children completed three doses of DTaP/IPV/Hib/HepB and 89.2% of 24 months olds were vaccinated with one dose of MMR (NHS Digital, 2022). Significant variation by area and population group as well as small declines in uptake each year since 2012 (NHS Digital, 2022) are causes for concern and leave considerable room for improvement to meet WHO vaccine uptake targets to prevent disease outbreaks. Once achieved, maintaining high uptake is vital and all opportunities to maximise vaccine uptake should be explored. As the primary administrators of infant vaccines in the UK, practice nurses (PNs) have a key role to play in managing vaccination pain.

### Evidence-based immunisation pain management

A clinical practice guideline on reducing pain at the time of vaccination was published in 2015 (Taddio *et al.*, 2015). The guideline was informed by systematic reviews of strategies to reduce the pain of immunisation and remains the best available source of evidence for paediatric immunisation pain management strategies. It includes interventions in five domains: physical, procedural, pharmacological, psychological and process (summarised in Table 1). The review was also used by WHO to develop global recommendations for pain mitigation at the time of vaccination in 2015, which WHO (2016) states should be viewed as an essential aspect of good practice worldwide. These interventions do not decrease the efficacy of vaccines, are effective and age-specific, and most require little or no additional time or resources (WHO, 2016). However, studies conducted in many countries (Taddio *et al.*, 2007; Harrison *et al.*, 2013; Harrison *et al.*, 2014) have shown that they are underused, and most people undergoing immunisation do not receive evidence-based interventions for pain management (McMurtry *et al.*, 2015). There is a lack of knowledge about the use of pain management interventions in infant vaccination in UK practice.

**Table 1.** Summary of recommendations for reducing pain during vaccine injections in infants – adapted from a 2015 clinical practice guideline (Taddio *et al.*, 2015)

Domain of pain management	Recommendation	Strength of recommendation
Procedural	No aspiration of needle	Strong
	Most painful vaccine last	Strong
Physical	Holding infant	Strong
Pharmacological	Topical anaesthetics before injection	Strong
	Sweet solutions before injection*	Strong
	Breastfeeding during injections	Strong
Psychological	Distraction	Weak
Process	Parent education	Strong
	Clinician education	Strong

* Sucrose solution or oral rotavirus vaccine.

### Aims

This study aimed to explore the use of evidence-based pain management strategies in infant immunisation among primary care PNs in the UK and to identify barriers to evidence-based practice.

## Materials and methods

### Design

A mixed methods design was used including a survey investigating PNs’ use of interventions and views on immunisation pain, and observations of infant immunisation appointments in London primary care vaccination clinics. Here, we report the findings from the survey. The study was approved by the University College London Research Ethics Committee.

### Questionnaire

A questionnaire was developed regarding pain management practices used by UK PNs in infant immunisation. Questions assessed the use of pain management interventions (based on current evidence (Taddio *et al.*, 2015)) as well as barriers to their use. Several intervention questions were adapted from a questionnaire used in a similar study (Harrison *et al.*, 2013). The questionnaire was reviewed by one expert in the field (HB) and then pilot-tested by 11 PNs who were invited to complete a paper version of the questionnaire during a national immunisation training course in London. Minor adjustments were made following feedback and analysis of responses.

The final questionnaire contained 24 questions (Appendix A). The first four questions collected demographic data. Eight questions were concerned with process interventions; three were about pharmacologic interventions; four regarded psychological interventions; and three were about physical/procedural interventions.

The frequencies with which PNs used these interventions were measured using a four-point Likert scale (‘never’, ‘occasionally’, ‘often’ or ‘always’); for each of these questions, if the response was ‘never’, the respondent was asked to provide further detail about their reason for this response to collect data regarding barriers to intervention use. Process intervention questions were generally ‘yes/no’ responses. Three questions addressed the evidence-practice gap. Respondents could also list additional pain management techniques they use, and space was provided for additional comments at the end of the questionnaire. A participant information sheet was included with the questionnaire, outlining the purpose, voluntary nature of participation, risks/disadvantages and possible benefits of the study. Contact information for the project supervisor was also provided. Informed consent was implicit in the completion and submission of the questionnaire.

### Participants and recruitment

Participants were recruited via convenience sampling. All PNs in the UK who administer childhood immunisations were eligible for inclusion; the recruitment methods reflect the aim of reaching as many eligible PNs as possible. The questionnaire was made available in both print and electronic (online) formats. The researchers distributed paper questionnaires in person to PNs attending several immunisation training courses in London; questionnaires were collected by the researchers upon completion. The online questionnaire was developed using Opinio survey software (Objectplanet, Inc., 2017). A link to the questionnaire was sent to nursing professionals in primary care, including immunisation leads, general PNs, immunisation trainers and university staff members. It was distributed between 16 June and 16 July 2017. The response rate cannot be estimated due to the nature of recruitment: the questionnaire link was available on various online platforms, including social media pages, websites and via email. Approximately 15 professionals were contacted and requested to distribute the questionnaire; 12 responded affirmatively and distributed the link within their practice areas.

### Data analysis

Data were analysed descriptively and summarised as frequencies (percentages) with 95% confidence intervals (CIs) when appropriate. Statistical significance tests were conducted to explore associations between use of pain management interventions and PNs’ characteristics (Table 2). Associations with *P*-values were calculated using Chi-squared and Fisher’s exact tests of independence for nominal variables. Mann–Whitney tests, Chi-squared tests and Spearman’s correlation coefficient were used for testing associations for ordinal variables. Data were managed and analysed using SPSS (IBM Corporation, 2009). A *P*-value < 0.05 was considered statistically significant. Conventional content analysis (Hsieh and Shannon, 2005) was used to analyse data from open-ended text responses regarding PNs’ reasons for never using interventions. Data were read repeatedly to derive codes, which were then labelled and ordered into an initial coding scheme; codes were then linked and sorted into themes, which are presented alongside the quantitative results.

**Table 2.** Variables tested in association testing

Use of pain management interventions	Practice nurse characteristics
• Parent/caregiver teaching • Injection method • Lap positioning • Breastfeeding • Topical anaesthetics • Sweet solutions • Nurse-led distraction • Parent/caregiver-led distraction	• Years of practice • Previous pain management training • Number of immunisations administered per week • Presence of pain management policy in practice area • Questionnaire format (online versus paper) • Interest in learning about pain management interventions

## Results

### Respondent characteristics

A total of 255 PNs practising in various regions of the UK completed the questionnaire (Table 3). The majority of respondents had been practising for more than 10 years and administered between five and 20 childhood immunisations per week.

**Table 3.** Characteristics of practice nurses

Characteristic	Number of respondents (%)
**Years of practice (** ***n*** ** = 254)**	
Less than 1 year	18 (7.1)
1 to 3 years	32 (12.6)
4 to 10 years	57 (22.4)
More than 10 years	147 (57.9)
**Number of immunisations administered per week (** ***n*** ** = 254)**	
Less than 5	20 (7.9)
5 to 10	100 (39.4)
10 to 20	86 (33.9)
More than 20	48 (18.9)
**Location (** ***n*** ** = 245)**	
North of England	46 (18.8)
Midlands and East of England	108 (44.1)
London	36 (14.7)
South of England	49 (20)
Scotland	4 (1.6)
Wales	2 (0.8)

### Interventions

#### Pharmacological and psychological

**Figure 1.**
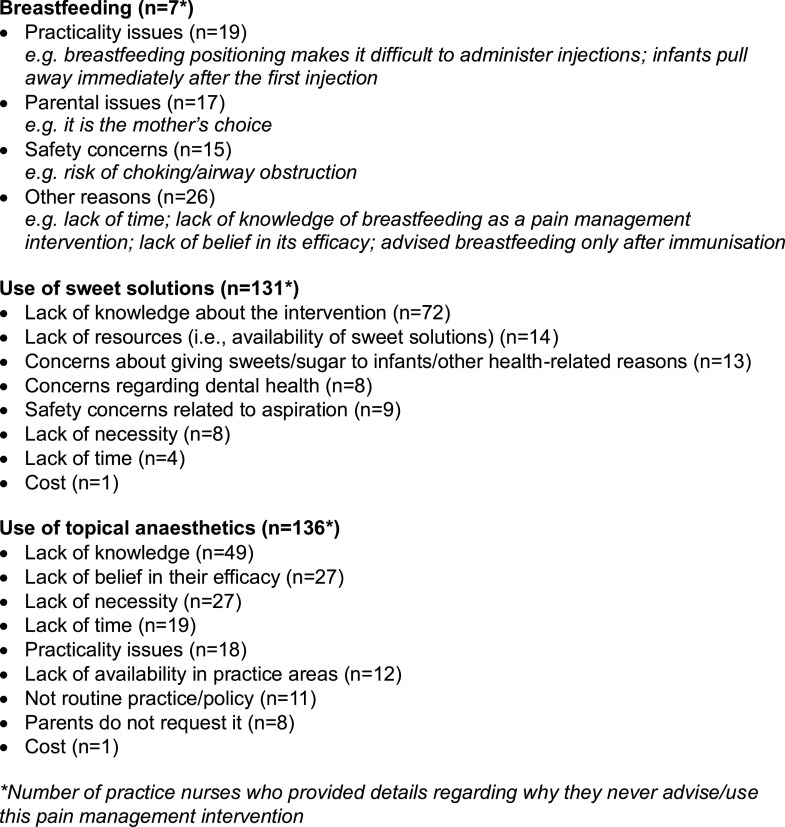
Reasons given by practice nurses for not advising breastfeeding, using sweet solutions or topical anaesthetics during infant immunisation

Table 4 summarises the frequency of use of pharmacological and psychological strategies used by PNs during infant immunisation. Of the 250 PNs who responded to these questions, 90.4% (CI 86.2%, 93.3%) reported never using topical anaesthetics or sweet solutions during immunisation. Responses regarding breastfeeding were more variable, with 41.9% (CI 35.9%, 48.1%) advising it occasionally (*n =* 103). The main reasons given for not advising breastfeeding, using sweet solutions or topical anaesthetics, are summarised in Figure 1. Lack of knowledge of the intervention, a belief it is not efficacious and lack of time were mentioned as reasons for not using all three interventions.

**Table 4.** Reported frequency of use of pharmacological and psychological strategies by practice nurses during infant immunisation

**Intervention (** ***n*** **=total responding)**	Reported frequency of use, % (*n* **)**			
	Always	Often	Occasionally	Never
**Pharmacological pain management strategies**				
Topical anaesthetics (*n* = 250)	0	0	9.6 (24)	90.4 (226)
Sweet solutions (*n* = 250)	0	3.2 (8)	6.4 (16)	90.4 (226)
Breastfeeding (*n* = 246)	6.5 (16)	17.5 (43)	41.9 (103)	34.1 (84)
**Psychological strategies**				
Parent-/caregiver-led distraction (*n* = 242)	6.6 (16)	30.6 (74)	47.9 (116)	14.9 (36)
Nurse-led distraction (*n* = 242)	12.0 (29)	25.2 (61)	38.0 (92)	24.8 (60)

Some PNs (*n =* 20) advised breastfeeding after the immunisations were given. Fifteen PNs reported administering the rotavirus vaccine first – this oral vaccine contains sucrose, but it is unclear whether this is the reason it was given first. Notably, a common reason given by PNs for not using topical anaesthetics is that vaccines are given by intramuscular injection, while topical anaesthetics work at skin level and therefore they considered they would not be effective in reducing pain.

##### Nurse-led distraction

The most frequently cited reason for not using nurse-led distraction techniques was practicality (*n =* 23), related to the difficulty of both administering injections and providing distraction when working alone. Other reasons were lack of time (*n =* 6), a belief by nurses that it would increase parental anxiety and lack of resources such as toys or music available for nurses’ use (*n =* 6).

##### Parent-/caregiver-led distraction

Reasons given for never using parent-led distraction related to nurses considering parents instinctively knowing what to do, or that PNs did not suggest distraction but considered it acceptable if parents chose to do it independently (*n =* 14). Lack of time (*n = 3)*, resources (e.g., bubbles) (*n =* 2) and practicality issues (*n =* 5) such as parents needing to concentrate on securely holding the infant were also cited.

#### Physical and procedural

Table 5 presents the findings regarding the use of physical and procedural strategies.

**Table 5.** Reported use of physical and procedural strategies by practice nurses during infant immunisation

Intervention	Number of respondents (%)
**Injection technique (** ***n*** ** = 228)**	
Rapid	167 (73.2 [CI 67.2%, 78.6%])
Slow	55 (24.1)
Aspiration	6 (2.6)
**Positioning of infant (** ***n*** ** = 255)**	
On parent/caregiver’s lap	255 (74.5)
Supine	15 (5.0)
Seated upright on chair	26 (8.6)
Other	13 (4.3)
**Order of vaccine administration (** ***n*** ** = 222)**	
Particular order	183 (82.4)
No particular order	39 (17.6)

##### Positioning

Respondents could select multiple responses regarding positioning. Those who selected ‘other’ (*n =* 13) provided descriptions such as: variations of lap positioning (*n =* 5), parent/caregiver cradling the infant (*n =* 2), responses about positioning being dependent on the child’s age (*n =* 3) and parental preference (*n =* 2).

##### Order of vaccine administration

Of the 183 respondents who reported administering vaccines in a particular order, 152 provided further detail. Some responses included giving the rotavirus vaccine first (21.7%, *n =* 33), although 5.9% (*n =* 9) gave it last; 9.2% (*n =* 14) reported giving the vaccine perceived to be most painful last (or giving those that were perceived as less painful first); and 7.2% (*n =* 11) involved simultaneous injection (involving an additional PN).

#### Process

##### Parent education

Sixty-eight per cent (CI 61.9%, 74.1%) of PNs reported regularly teaching parents about pain management interventions (*n =* 151).

##### Immunisation pain management policy

Most respondents (81.1%, *n =* 184) reported that they did not have a pain management policy for immunisation in their practice area; 4.4% (*n =* 10) reported their practice area did have one; and 14.5% (*n* = 33) were unsure.

##### Health professional education

All respondents reported attending immunisation training, but only 13% (*n =* 28) of PNs reported that this had included pain management. However, 93% (CI 88.8%, 95.7%, *n =* 200) of PNs reported an interest in learning about pain management strategies for infant immunisation.

### Challenges associated with the use of pain management strategies

The main challenges reported were lack of knowledge of evidence-based interventions (60% *n* = 153), lack of time (53.7% *n* = 136) and lack of resources (47.1% *n* = 120). Two PNs considered pain management interventions to be unnecessary.

### Associations

There was a statistically significant association between previous pain management training and parent/caregiver teaching, χ^2^ (1) = 7.258, *P* = 0.007, while 92.3% of PNs with previous training reported regularly teaching parents about pain management, only 66.3% of those without previous training taught parents about pain management. Additionally, a statistically significant association was observed between years of practice and parent teaching: PNs with more years of experience were more likely to teach parents about pain management compared with those with fewer years of experience (χ^2^ (3) = 8.640, *P* = 0.034).

## Discussion

To our knowledge, this is the first study conducted among UK PNs of their strategies to manage pain during infant vaccination. We found these strategies are not frequently utilised, and multiple barriers to evidence-based practice were reported relating to lack of time, knowledge and resources.

Consistent with previous research (Harrison *et al.*, 2013), we found that distraction techniques were used more frequently than any other intervention, particularly parent-/caregiver-led distraction. Harrison’s Australian study analysing immunisation practices found 71.8% of infant immunisations involved caregivers distracting the infant by singing, talking or with toys (Harrison *et al.*, 2013). Similarly in Canada, Taddio *et al.* (2007) found 97% of paediatricians and 92% of mothers reported using non-pharmacological pain reduction techniques including distraction; however, this also included other strategies such as holding the infant. In the current study, approximately 85% of PNs reported positioning the infant on the parent/caregiver’s lap. This contrasts with findings from the Harrison *et al.* (2014) study in which 62% of YouTube videos showed infants being placed supine during immunisation.

Our finding that PNs did not consistently advise breastfeeding during immunisation is also consistent with Harrison *et al*.’s study (2013). Although it is unknown whether post-vaccination breastfeeding reduces pain (Taddio *et al.*, 2015), we found it was frequently recommended by PNs after administration of injections.

Sweet solutions were even less likely to be used with 90.4% (*n =* 226) of respondents reporting they never used them. However, in this study, almost a quarter of PNs who reported administering vaccines in a particular order administered the rotavirus vaccine first (*n =* 33), which contains sucrose and could therefore be used as an alternative (though it is unknown whether pain reduction was their reason for administering vaccines in this order). The other evidence-based strategy reported by 9.2% (*n =* 14) of PNs in relation to order of administration was administering the most painful vaccine last. Order of vaccine administration has not been assessed in previous studies of this nature. Similar to findings regarding the use of sweet solutions, 90.4% of PNs reported never using topical anaesthetics (*n =* 226). This is also higher than in previous studies: Harrison *et al*. (2013) found 81.1% of PNs never use these medications, while Taddio *et al.* (2007) found 26.2% and 61.9% of paediatricians never or rarely use them, respectively.

Almost 70% of PNs reported teaching parents about immunisation pain management strategies, a figure similar to that reported by Taddio *et al.* (2007) although that study focused on paediatricians. This is of interest in view of the reported underutilisation of strategies by the nurses themselves. However, Taddio also reported inconsistency between paediatricians’ and parents’ reports of such counselling, with only 42% of surveyed mothers reporting receiving it, raising the possibility of inaccurate self-report of practices in our study.

Most PNs reported that their practice area did not have a pain management policy for immunisation (81.1%, *n =* 184) which may suggest a lack of support or investment at policy level regarding immunisation pain management. The presence of a pain management policy has been shown to increase the use of evidence-based interventions, and prioritisation of a particular health care practice at the institutional level has been shown to improve that practice more effectively than simply targeting the individual’s behaviour (i.e., the nurse) (Reis *et al.*, 2003). Pain management strategies not being routine practice/policy were identified frequently by the PNs in our study as a reason for not using these strategies, highlighting the importance of having such policies in place. For example, 11 PNs reported this as their reason for never using topical anaesthetics, using phrasing such as ‘*not our protocol’* or ‘*not usual practice’*. This represents a different type of barrier to evidence-based practice that must be targeted at the policy level and could be addressed by the establishment of pain management policies within practice areas.

Previous studies have shown that parents believe it is health professionals’ responsibility to make immunisation less painful (Taddio *et al.*, 2012). In contrast, many PNs in this study reported the reason they never used certain interventions was because the parents had not requested them (e.g., regarding breastfeeding: ‘*mums have never asked’*, or sweet solutions: ‘*parents have never attended with any sweet solutions’*). These responses imply that it is the parent’s responsibility to manage pain, rather than the nurse’s.

Reasons for never using each intervention varied between domains of pain management; however, some themes were common throughout. Lack of time, lack of knowledge, lack of resources, issues of practicality, and the belief that strategies were unnecessary, or nurses should ‘get on with it’ were noted across interventions. Taddio *et al.* (2007) reported similar reasons for not using topical anaesthetics to those identified in this study (i.e., time, lack of parental request, cost and institutional practices). Paediatricians in their study also listed reasons not identified in this study such as personal experience, discussion with colleagues and parental refusal. This contrast in findings may be due to a difference in data collection methods (i.e., questionnaire phrasing/design), as well as professional differences, such as the scope of practice of paediatricians and nurses or differences in the Canadian and UK health care systems. A focus group study investigating nurses’ perceptions on immunisation pain throughout childhood found similar barriers including lack of knowledge and resources (Kikuta *et al.*, 2011). Previous studies have not assessed health professionals’ interest in learning about pain management for childhood immunisation; however, one of the main challenges identified by PNs in this study was a lack of knowledge of interventions. As motivation is a factor in reducing the evidence–practice gap, an important finding in this study is that 93% (*n =* 200) of PNs expressed an interest in learning about pain management techniques.

Some misconceptions about pain relief were evident, for example although breastfeeding is considered a safe and effective intervention (Taddio *et al.*, 2009), with choking not reported among infants who breastfeed during vaccination, some respondents (*n =* 15) who never advised it listed concern about choking as their justification. Another common reason for never using interventions was a lack in belief in their efficacy. Similar attitudes have been previously reported among nurses (Kikuta *et al.*, 2011). Taddio *et al.* (2009) explored myths about immunisation pain and found similar results, including beliefs that pain is not an issue for children and analgesic creams do not work. These findings demonstrate that the concept of ‘lack of knowledge’ goes beyond a straightforward lack of awareness of interventions; clarifying misconceptions about immunisation pain management represents another opportunity to improve practice.

We found that PNs whose previous immunisation training had included pain management reported regularly teaching parents more frequently than PNs whose training had not included it. In the United States, the introduction of a clinical protocol for nurses proved to be an effective tool to introduce breastfeeding as a method for relieving the pain of vaccination (Komaroff and Forest, 2020).

In the UK, despite the availability of standards and a curriculum for immunisation training and e-learning materials (Public Health England, 2018; Health Education England, 2023), only brief information was added to the latter resource in 2020 about strategies to reduce the pain and distress of vaccination in infants. The ‘Green Book’, UKHSA’s principal publication containing information on vaccines and immunisation procedures, also does not include any specific information about pain management (UKHSA, 2022) but does include advice not to aspirate the syringe after inserting the needle. In response to the COVID-19 vaccination programme, some resources describing distraction techniques to reduce anxiety during vaccination were developed, for example by Public Health Wales (2021), but as the COVID-19 vaccination programme did not include infants, this advice is specific to older children. This minimal guidance may contribute to the underuse of these strategies.

The findings of this study contribute to the existing literature by demonstrating a potential evidence–practice gap in the UK similar to gaps previously identified in other countries and research. This is important, as it offers a more regionally specific understanding of current UK practice, but also because each study setting is unique in its immunisation training standards, policies and procedures. The findings of this study can be added to comparisons across settings, and patterns in practice and barriers may be identified and addressed. The findings also provide insight into the barriers to evidence-based practice, which facilitate a more comprehensive understanding of reasons why pain management interventions are not being used, and expose the aspects of practice to target in order to facilitate improvement most effectively.

### Limitations

Convenience sampling was conducted to achieve the greatest possible number of responses. Due to these recruitment methods, the sample was not randomly selected, nor is it necessarily representative of practices across the UK population of immunisers. Findings are therefore not necessarily generalisable, and results (including CIs) must be interpreted with this taken into consideration. Findings may represent the views of people particularly interested in immunisation. If this bias is present and participants were those with a particular interest, other non-participants may be even less aware of/likely to use pain management interventions; the conclusion that interventions are underused would remain likely. Although these data were collected in 2017, we consider it unlikely that there has been a widespread adoption of pain management strategies in the intervening years and no further UK research on this topic has been published in that time frame. This is supported by the findings of the 2022 UKHSA parental survey which revealed a significant proportion of those parents dissatisfied with their most recent vaccine visit were concerned about the pain and distress of vaccination (UKHSA, 2023).

The questionnaire link was available on Twitter and Facebook groups, which were related to immunisation, primary care/general practice nursing, etc. Nurses who were not active on social media or frequent users of the Internet were less likely to be represented; to combat this bias, the questionnaire was also distributed at immunisation training courses. There may still be non-response bias in who completed the questionnaire at these venues; due to the method of distribution, response rate cannot be calculated, and it is unknown how responders differ from non-responders. It is important to note that annual immunisation training is a requirement for all PNs in the UK: training is not attended solely by those with a special interest in immunisation (although respondents may have had a particular interest). Questionnaire results must also be considered acknowledging the presence of self-report bias: respondents may have aimed to present themselves positively, reporting socially acceptable practices. Therefore, pain management strategies may be used even less frequently than reported. Lastly, although barriers to evidence-based practice were explored in the questionnaire, failure to identify a barrier in this study does not necessarily mean it does not exist; further research is required to determine other barriers.

### Recommendations for practice and future research

This study found that lack of knowledge, time and resources were key barriers to intervention use. Pain management in infant immunisation could be improved through educational approaches for both practitioners and parents. In Canada, providing postnatal parents with information about interventions to reduce pain associated with immunisation, while in hospital was found to increase their use (Taddio *et al.*, 2018), an approach which would need testing in the UK setting. Education for immunisers about managing vaccination pain should be added to the immunisation training curriculum, with information made available in the ‘Green Book’ (UKHSA, 2022) and policy and procedure manuals.

## Conclusions

This study demonstrates that evidence-based pain management strategies are infrequently used during infant immunisation in the UK and provides evidence regarding barriers to intervention use. This evidence–practice gap results in unmitigated pain at the time of immunisation, a source of distress for infants and parents/caregivers and potential impediment to vaccine uptake. Most of the strategies explored in this study require minimal or no additional resources or time to implement. Efforts must be made to improve this aspect of immunisation practice and reduce infants’ pain and distress, which may also have an impact on improving future rates of vaccine uptake.

## Supplementary material

For supplementary material accompanying this paper visit http://doi.org/10.1017/S146342362300066X. click here to view supplementary material
