# Supplementary figures and images for: Pain management in infant immunisation: A cross-sectional survey of UK primary care nurses
Source: Prim Health Care Res Dev. 2023 Dec 21;24:e71. doi: 10.1017/S146342362300066X (PMC10790675; doi:10.1017/S146342362300066X)

**Supplementary Material**

1. Pages 2-6: Appendix A: Questionnaire

**Appendix A: Questionnaire**


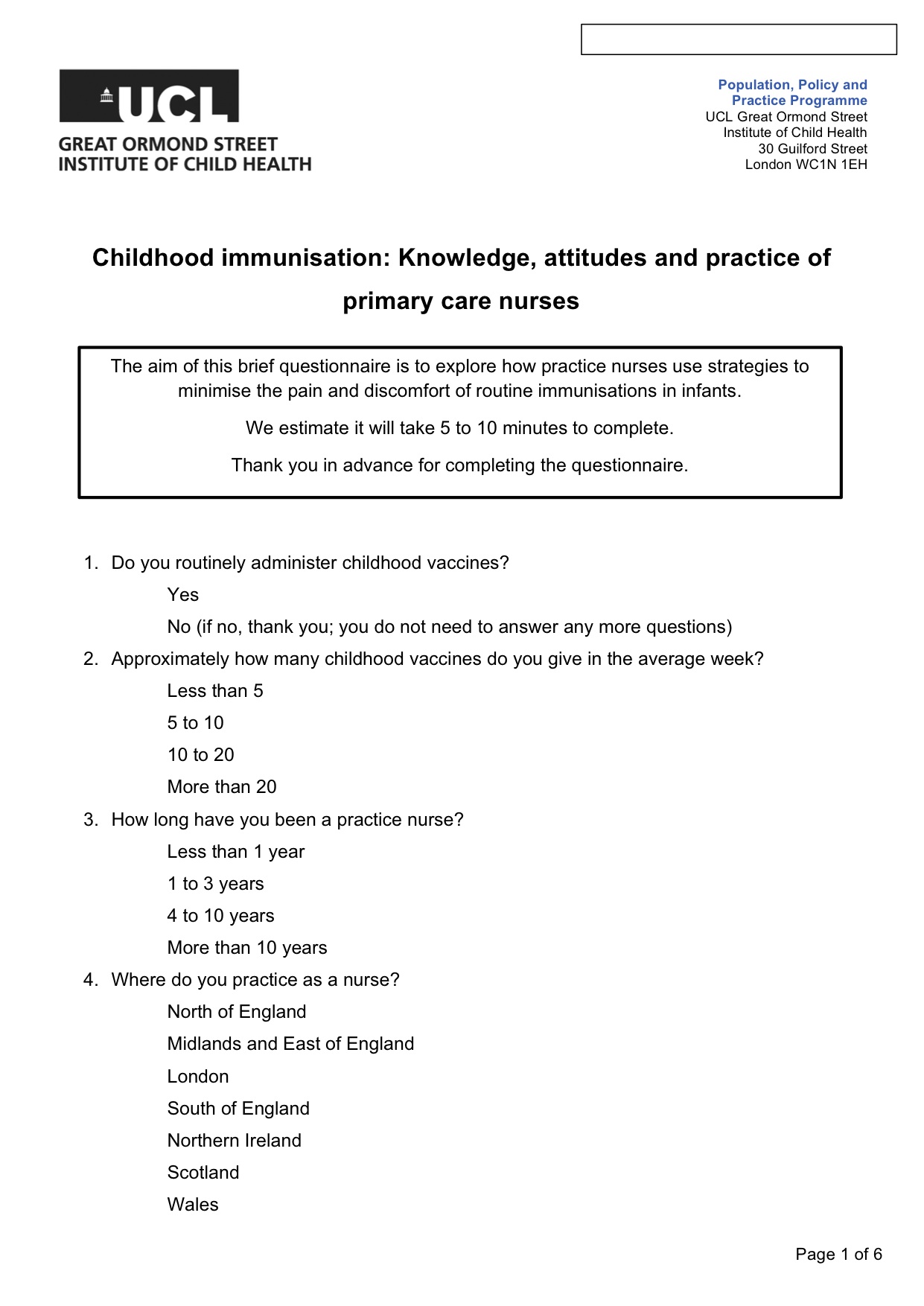


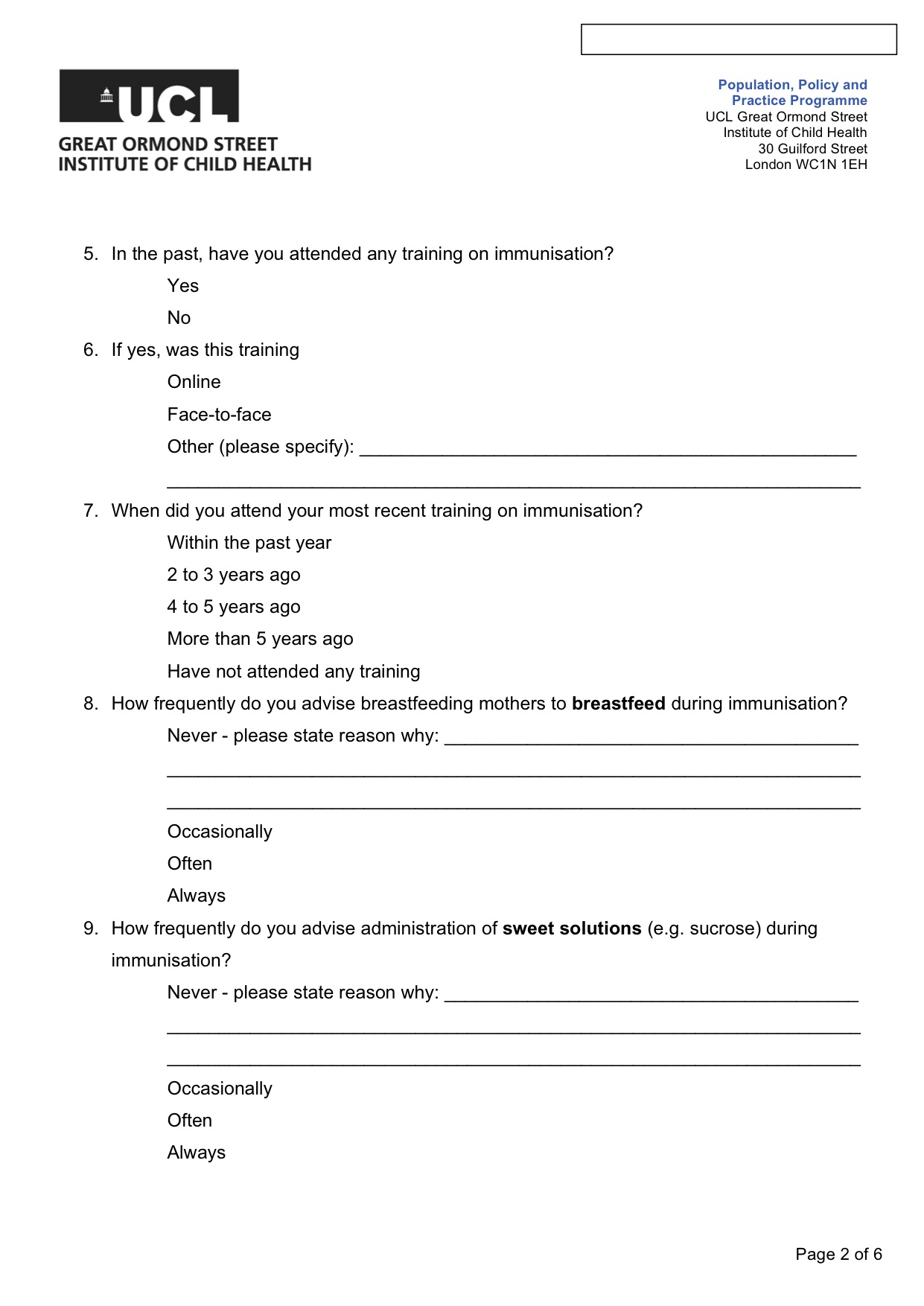


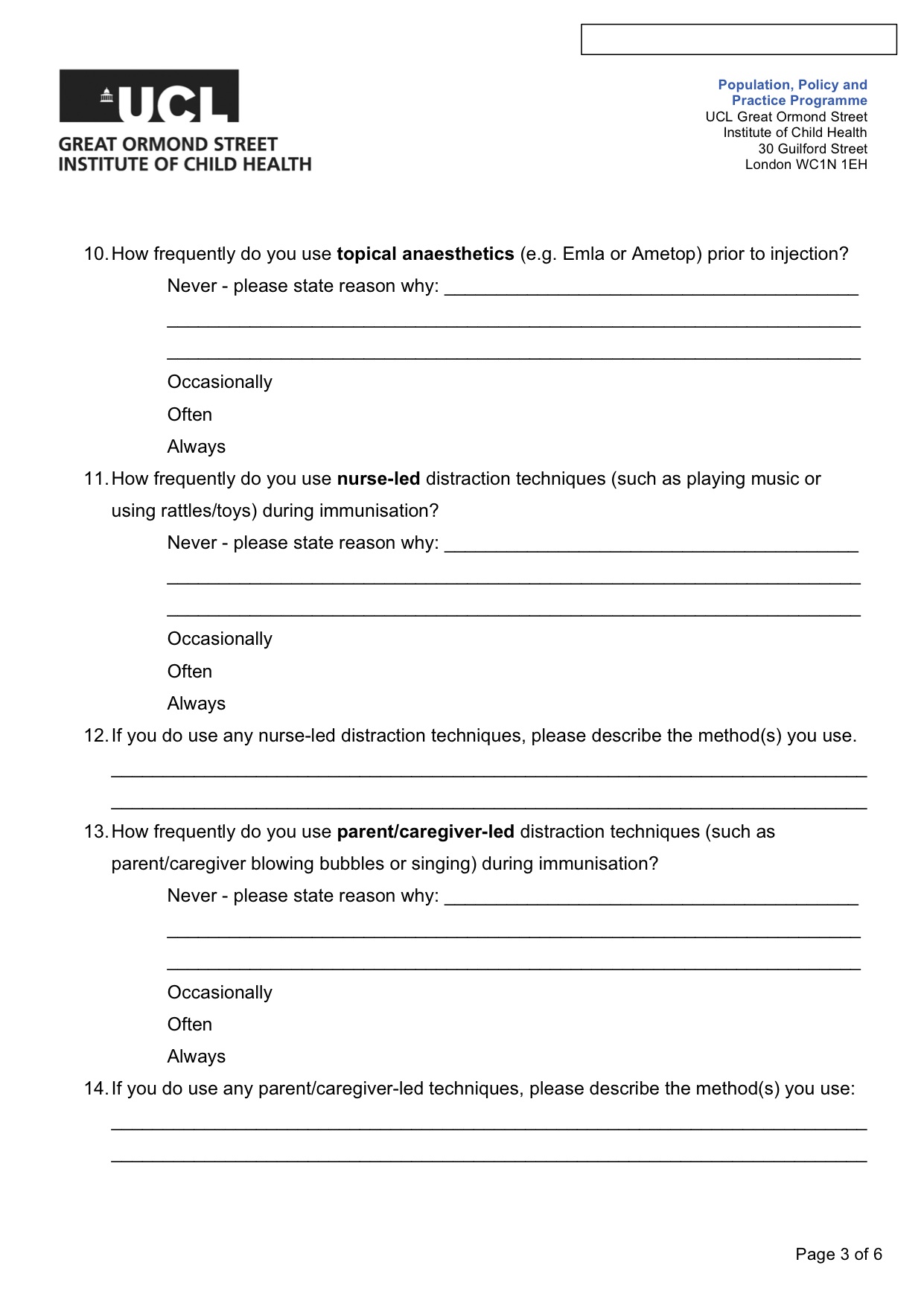


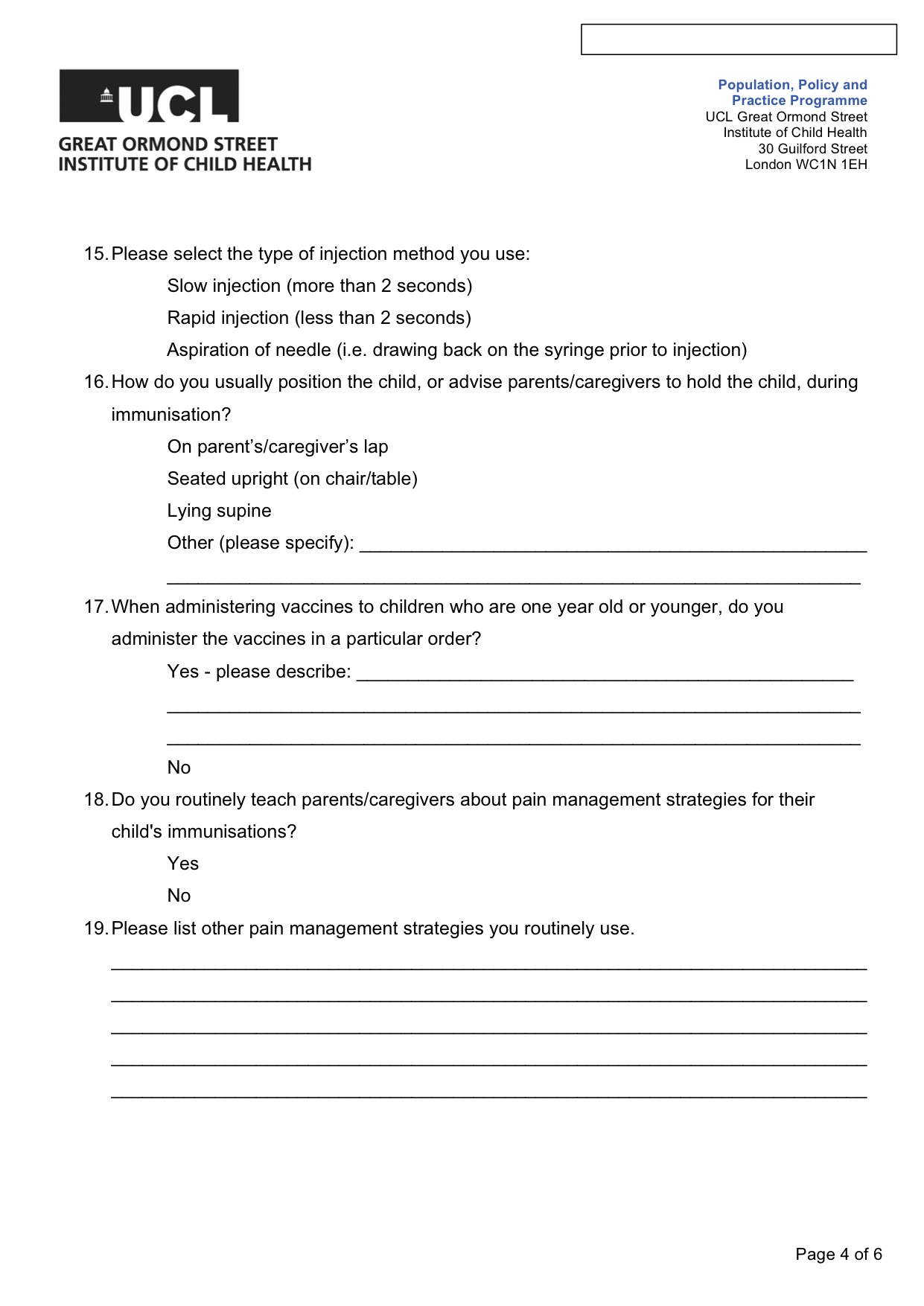


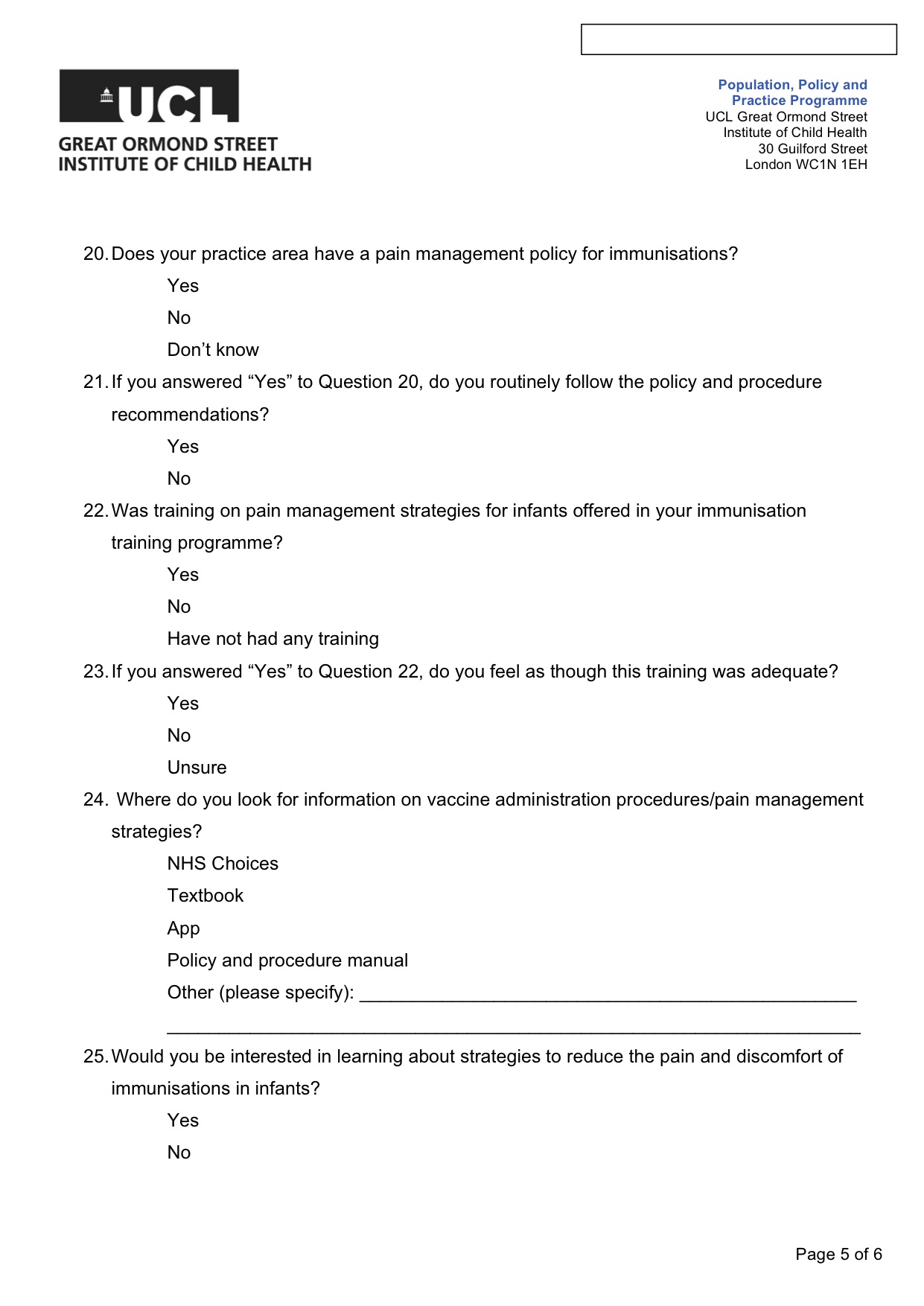


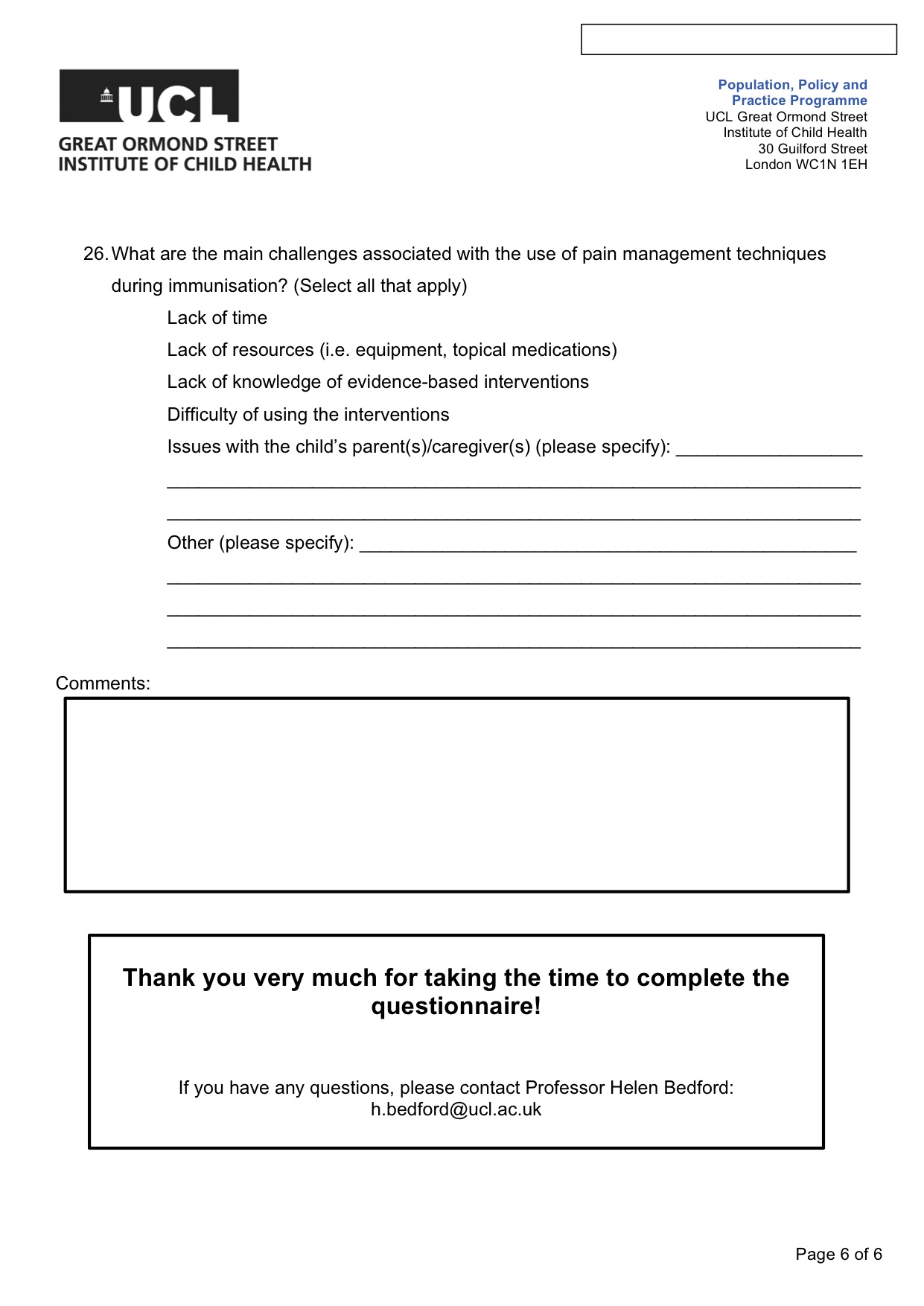

Supplement: Supplementary file 1 [file S146342362300066Xsup001.docx]
